# Acupuncture to Improve Patient Discomfort During Upper Gastrointestinal Endoscopy: Systematic Review and Meta-Analysis

**DOI:** 10.3389/fmed.2022.865035

**Published:** 2022-06-03

**Authors:** Ning Gao, Huan Chen, Yang Wang, Yufeng Guo, Zhishun Liu, Weiming Wang

**Affiliations:** ^1^Department of Acupuncture and Moxibustion, Guang'anmen Hospital, China Academy of Chinese Medical Sciences, Beijing, China; ^2^School of Acupuncture-Moxibustion and Tuina, Tianjin University of Traditional Chinese Medicine, Tianjin, China

**Keywords:** acupuncture, endoscopy, gastrointestinal, systematic review, meta-analysis

## Abstract

**Background and Aims:**

Severe discomfort during an upper gastrointestinal endoscopy (UGE) is often a stressful experience for patients undergoing the procedure. An increasing number of studies have shown that acupuncture may reduce discomfort during UGE. A systematic review in 2004 investigated the effect of acupuncture for gastrointestinal endoscopy, but these data have not been recently reviewed. Therefore, this study was conducted to evaluate the current evidence and provide up-to-date knowledge for clinical decision-making.

**Methods:**

Nine databases were searched from inception to June 2021. Eligible randomized controlled trials (RCTs) were included. The outcome data were synthesized where necessary, and risks of bias of included studies were assessed using RevMan V.5.3.

**Results:**

Twenty-three eligible RCTs with 3,349 patients were identified. It was found that acupuncture plus topical pharyngeal anesthesia with lidocaine hydrochloride (TPALH) resulted in greater improvements regarding visual analog scale (VAS) scores and the incidence of nausea and vomiting (INV) when compared with TPALH alone. These results were consistent among studies of manual acupuncture, electroacupuncture, auricular-plaster, superficial needle (SFN) and acupressure. In the meta-analysis, SFN plus TPALH showed significant improvement of VAS scores compared to sham SFN plus TPALH (MD −1.11, 95% CI −1.52 to −0.70, *P* < 0.00001). Most of included studies did not report any side effects in their findings, and were of medium-to-high risk of bias.

**Conclusion:**

Acupuncture, as adjunctive therapy to TPA, may result in less patient discomfort than TPA alone. Findings from this review should be interpreted with caution due to the high heterogeneity identified. There is low-quality evidence supporting the use of acupuncture over sham. More rigorously designed RCTs are needed to inform clinical decision-making.

**Systematic Review Registration:**

PROSPERO [CRD42014008966].

## Introduction

Severe discomfort due to strong gag reflexes and pain during upper gastrointestinal endoscopy (UGE) often results in a stressful experience for patients who undergo the procedure and occasionally hinders the success of the procedure (1, 2). As a result, sedated UGE procedures with less discomfort and pain have been the predominant method used in endoscopic clinics in Europe and North America (3, 4). However, there are concerns regarding the cost and adverse events (e.g., cardiopulmonary events, allergic reactions) associated with the use of sedatives for UGE, especially in the elderly population with pre-existing cardiopulmonary disease (5, 6). Therefore, unsedated UGE is still being used by many physicians and patients in China and other developing countries (7, 8). Topical pharyngeal anesthesia (TPA), which has been reported to be effective in suppressing the threshold of the gag reflex, is often applied before an unsedated UGE to ease discomfort and pain (9–11). However, involuntary gagging cannot be suppressed among certain patients even after the use of TPA due to sensitive gag reflexes (9).

Acupuncture is a therapeutic intervention that involves the insertion of fine needles into the skin or deeper tissues at specific locations on the surface of the body with the aim of curing disease or promoting health, according to the theory of Traditional Chinese Medicine (12). Acupuncture has been frequently used to treat various diseases including nausea and vomiting associated with chemotherapy, pregnancy, and recovery from surgical procedures (13, 14), and some published studies have also demonstrated that acupuncture may be able to increase tolerance and reduce discomfort during UGE (15, 16). A systematic review in 2004 on the effect of acupuncture during gastrointestinal (GI) endoscopies included only six studies with inconclusive findings. However, it did not distinguish UGEs from colonoscopies, nor sedated from unsedated procedures, during which the patient status would be very different (17). On the other hand, the number of studies focusing on acupuncture to relieve patient discomfort during an unsedated UGE has increased, and many have reported that acupuncture was often used in conjunction with TPA during an unsedated UGE. However, there have been no systematic reviews concerning the effect of acupuncture on discomfort during UGE since 2004. Therefore, the current systematic review and meta-analysis was conducted with the aim of evaluating current evidence on acupuncture for the management of discomfort during an unsedated UGE, and thus providing up-to-date recommendations for clinical practice and decision-making.

## Methods

This systematic review and meta-analysis were conducted according to the Preferred Reporting Items for Systematic Reviews and Meta-Analyses (PRISMA) (18). The protocol was registered at PROSPERO with registration number CRD42014008966 (19).

### Search Strategy

The following databases were searched from inception to June 2021: MEDLINE, EMBASE, the Cochrane Central Register of Controlled Trials, Scopus, Web of Science, the Chinese Biomedical Literature Database, the China National Knowledge Infrastructure, Wanfang Database, VIP Database, the WHO International Clinical Trials Registry Platform portal, and ClinicalTrials.gov. The key search terms included: “endoscopy,” “upper gastrointestinal endoscopy,” “discomfort,” and “acupuncture,” etc. Tailored search strategies were developed for each database. Published review papers were searched to identify additional references.

### Inclusion Criteria

Studies were included if they focused on (1) Population: patients who received an unsedated UGE (e.g., screening, surveillance, diagnosis; without the limitation of the brands or models of gastroscopes), regardless of age, sex, or race; (2) Intervention: were evaluating either invasive or non-invasive acupuncture therapies with or without concomitant treatment, with the aim of relieving discomfort during UGE (acupuncture hereby was defined as any treatment methods that achieve their effect by stimulating acupoints on body, including electroacupuncture, manual acupuncture, acupressure, etc.); (3) Comparison: were comparing acupuncture with any conservative interventions, not limited to the following: no treatment, placebo, sham acupuncture (SA), or other active conservative interventions (e.g., lubricant use, TPA, and sedation); and (4)Outcomes and Studies: were RCTs reporting at least one of the following outcomes, including discomfort severity using validated scales [e.g., visual analog scale (VAS), numerical rating scale (NRS)], incidence of nausea and vomiting (INV) during the UGE procedure, the proportion of patients satisfied with the process or patients who would opt for the same procedure again, and the incidence and types of adverse events related to acupuncture treatment regardless of language.

### Exclusion Criteria

Studies were excluded if they (1) were investigating patients having chronic pharyngolaryngitis, severe digestive system diseases, persistent hiccups, severe nausea and retching, proven tumors in the upper digestive tract, severe mental disorders, or uncontrolled cardiopulmonary disease; (2) were only comparing different types of acupunctures without a comparison group of no treatment, placebo or sham acupuncture, medicine, or other conservative therapies; and (3) were not RCTs or were quasi-RCTs, or without a clear description of interventions, or did not provide outcome data.

### Study Selection and Data Extraction

Two reviewers (Ning Gao and Huan Chen) independently reviewed all retrieved papers by title and abstract to identify relevant papers, then the full texts of relevant papers were retrieved and reviewed for eligibility according to inclusion and exclusion criteria. Data were then extracted from the included studies, including author and year of the study, patient characteristics, study design, sample size, treatment type and regimen of experiment, control groups, outcomes measures, etc. Disagreements were resolved *via* discussion or arbitration by a third reviewer if necessary.

### Assessment of Risk-of-Bias

According to the “risk-of-bias” tool from the Cochrane Handbook for Systematic Reviews of Interventions, two reviewers independently evaluated the risk of bias for the included studies considering the following seven domains: random sequence generation, allocation concealment, blinding of patients and personnel, blinding of outcome assessment, incomplete outcome data, selective outcome reporting, and other sources of bias (20). Each domain was rated as “low risk,” “high risk,” or “unclear risk.”

### Data Analysis

All studies were categorized based on the types of interventions. For continuous variables (e.g., VAS), the mean difference (MD) with standard deviation was used to present treatment effect. For dichotomous variables (e.g., INV), treatment effects were presented as a risk ratio (RR) with 95% confidence intervals (CIs).

Outcome data were synthesized to estimate the pooled effect size of acupuncture where applicable. The heterogeneity across studies would be assessed using the *I*^2^ and the chi-square tests and was considered significant at *I*^2^ > 50% or *P* < 0.1. A random-effects model was used if heterogeneity was significant, otherwise a fixed-effects model was used. Sensitivity analysis was conducted by removing a single study to explore if the influence of each study would change the direction of the pooled effect size in the meta-analysis.

## Results

**Figure 1:**
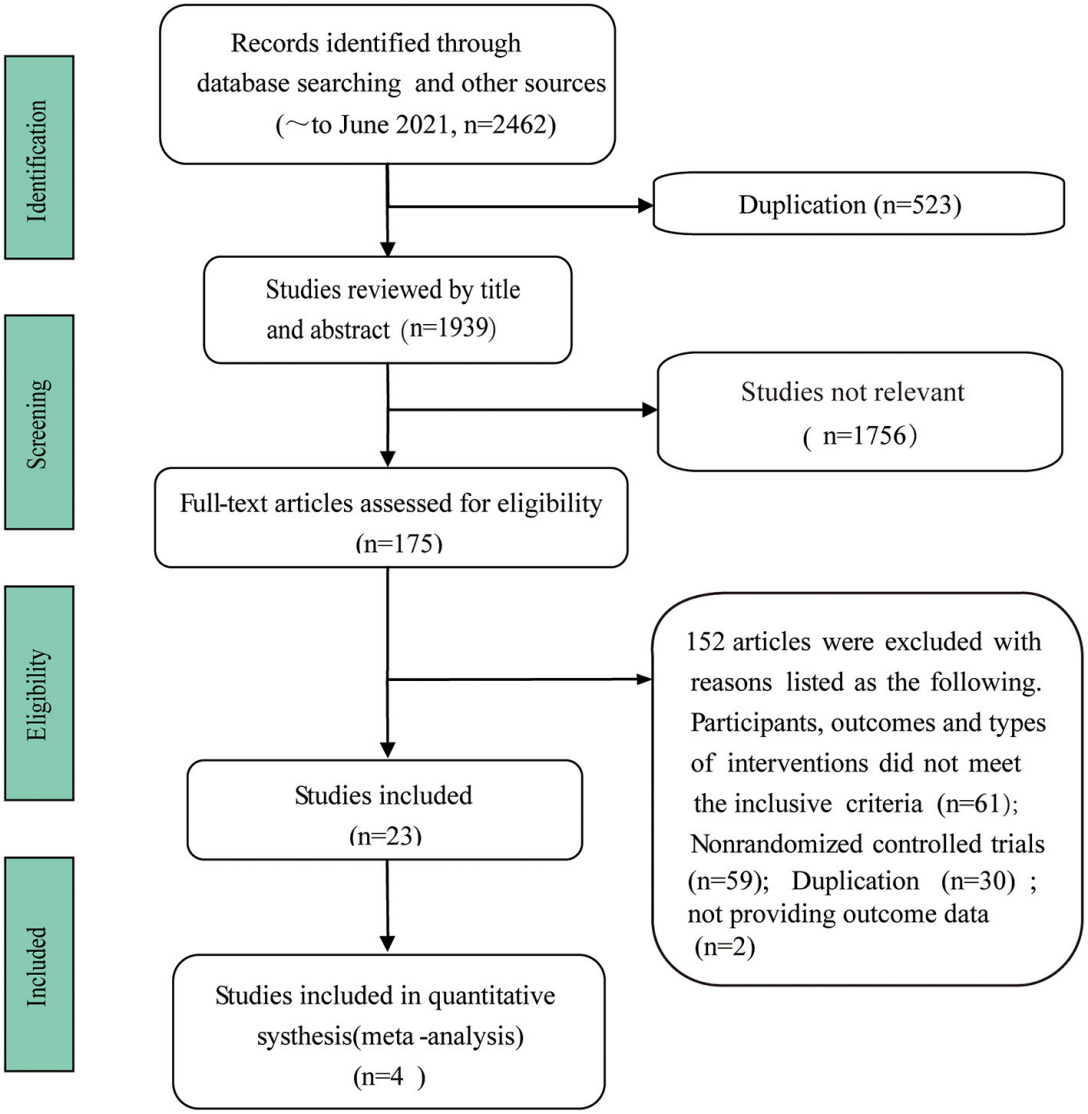
Flow diagram of study selection process.

A total of 2,462 studies were identified through an initial search. After removing duplicates, 1,939 studies were reviewed by title and abstract, and 1,756 studies were excluded for not meeting the inclusion criteria. Next, the full-text of 175 studies were obtained for further assessment, and 23 studies were considered eligible for the review according to the inclusion and exclusion criteria, and four studies were included in the meta-analysis. The details of the study selection process were shown in a PRISMA flow diagram (Figure 1).

### Characteristics of the Included Studies

The 23 RCTs included were conducted in Germany (one study) (21), Turkey (one study) (15), France (one study) (16), and China (20 studies), (22–41). A total of 3,349 patients (1,717 male and 1,393 female) who underwent UGE were included, with ages ranging from 16 to 86 years. Two studies did not report the number of male and female patients included (16, 34).

Among the 23 RCTs, seven studies used an electronic gastroscope and one study used a fibergastroscope, while 15 studies did not report the type of gastroscope used. Studies were categorized by types of acupuncture assessed, including electroacupuncture (EA, five studies), manual acupuncture (MA, 10 studies), auricular plaster therapy (AP, two studies), superficial needle (SFN, two studies), acupressure (one study), transcutaneous electric nerve stimulation (TENS, one study), and a combination of EA and AP (two studies). The most frequently used acupoints were PC-6 (Neiguan) and LI-4 (Hegu) on the hands, and ST-36 (Zusanli) on legs based on the symptoms presented during UGE.

Twenty-one studies initiated the acupuncture treatment prior to the UGE procedure and continued treatment throughout the procedure, while two studies only applied acupuncture before the procedure. The average duration of acupuncture treatment was in accordance with the duration of the UGE procedure and varied across patients and performers.

In terms of outcome reporting, 14 studies reported INV observed by the researcher, eight studies reported the VAS scores evaluated by patients to assess discomfort, and eight studies reported the proportion of patients satisfied with the entire process or those willing to undergo the procedure again. The VAS scores were evaluated by participants right when UGE had finished, and INV were observed by researcher according the signs of participants during the whole procedure. Four studies reported the incidence and types of adverse events related to acupuncture treatment. Some studies (15, 16, 21) also reported other outcomes, such as number of intubation attempts and eructation, the rate of successfully performed procedures, anxiety scores, etc. The details of the included studies were summarized in Tables 1, 2.

**Table 1:** Characteristics of included studies.

**References**	**Country**	**Sample size (dropouts)**	**Age, mean ±SD Experiment/control**	**Interventions**	**Time point (T/C)**	**Types of gastroscopy**	**Regimens**	**Outcomes**
							**Dosage of TPALH**	
Wang et al. (28)	China	60 (0)30/30	G1: 45.37 ± 4.63	G1: MA + TPALH	5 min pre-operation to the end of procedure	NM	PC-6; with hand manipulating for the whole procedure	1. INV
			G2: 47.92 ± 7.28	G2: TPALH	5 min pre-operation		lidocaine 2% gel 5 ml	
Chen (29)	China	60 (0)30/30	G1: 49.57 ± 11.52	G1: SFN + TPALH	5 min pre-operation to the end of procedure	Olympus normal lens	PC-6; with hand manipulating for 2 min	1. INV
			G2: 48.63 ± 11.61	G2: SA + TPALH	Pre-operation		NM	2. vas of discomfort
								3. willingness to repeat the procedure
Qi (27)	China	80 (0)40/40	G1: 52.40 ± 12.26	G1: AP + TPALH	20 min pre-operation to the end of procedure	Pentax2970 *D* =9 .8mm	TF4,AH6,CO4,AT4,TG3; with hand manipulating ear beans for 20 min	1. vas of discomfort
			G2: 52.15 ± 12.95	G2: TPALH	Pre-operation		NM	2. willingness to repeat the procedure
Jiang (30)	China	156 (0)77/79	G1: 20–70	G1: acupressure + TPALH	2 min pre-operation to the end of procedure	NM	PC-6	1. INV
			G2: 22–68	G2: TPALH	10 min pre-operation		Lidocaine 2% gel twice	
Chen et al. (26)	China	97 (0)52/45	G1: 31.59 ± 6.98	G1: EA + TPALH	20 min pre-operation to the end of procedure	NM	LI-4, PC-6, ST-36	1. INV
			G2:31.60 ± 7.18	G2: TPALH	10 min pre-operation		Lidocaine gel 10 ml	2. willingness to repeat the procedure
Cui (22)	China	137 (3)66/68	G1:55.48 ± 6.64	G1: EA + TPALH	20 min pre-operation to the end of procedure	NM	ST-36	1. INV
			G2:55.91 ± 7.02	G2: TPALH	10 min pre-operation		Dicaine 0.2% spray three times	2. adverse effects
Zhang et al. (41)	China	160 (0)80/80	48(20–70)	G1: MA	Whole duration of the operation	NM	ST-36, PC-6	1. INV
				G2: TPALH	15 min pre-operation		Lidocaine 2% spray 1 ml	
Tian and Wu (38)	China	90 (0)50/40	G1:52.44 ± 9.51	G1: MA	10 min pre-operation to the end of procedure	NM	ST-36,PC-6; with hand manipulating at a interval of 2–3 min	1. INV
			G2: 47.25 ± 11.35	G2: no treatment	–		–	
Wang (40)	China	300 (0)169/131	43.6(23–60)	G1: MA	40–50 min pre-operation to the start of procedure	NM	PC-6; with hand manipulating at a interval of 10–15 min	1. INV
				G2: TPALH	15–20 min pre-operation		Lidocaine 2% spray three times	
Zhou et al. (35)	China	80 (0)40/40	G1: 34 ± 15	G1: MA + TPALH	Whole duration of the procedure	Electronic gastroscope	ST-36, PC-6	1. INV
			G2: 40 ± 18	G2: TPALH	10 min pre-operation		Lidocaine 2% gel 3 ml	
Zhou and Fang (23)	China	248 (6)123 (3)/125 (3)	G1: 41.93 ± 10.56	G1: EA + TPALH	3-5 min pre-operation to the end of procedure	NM	ST-36,PC-6	1. vas of discomfort
			G2: 39.90 ± 11.08	G2: TPALH	5 min pre-operation		Lidocaine 2% gel 5 ml	
Wu and Ye (32)	China	100 (0)50/50	G1:41.58 ± 13.15	G1: AP + EA + TPALH	15 min pre-operation to the end of procedure	NM	LI-4, ST-36, PC-6, TF4, AH6, CO4	1. INV
			G2:42.45 ± 12.76	G2: TPALH	15 min pre-operation		Lidocaine gel 10 ml	2. willingness to repeat the procedure
Wang et al. (49)	China	108 (0)54/54	G1: 51.74 ± 13.45	G1: MA + TPALH	10 min pre-operation to the end of procedure	NM	ST-34	1. vas of discomfort
			G2: 52.25 ± 12.16	G2: TPALH	10 min pre-operation		Lidocaine gel 10 ml	
Li and Wang (24)	China	98 (0)49/49	G1: 50.3 ± 3.8	G1: MA + TPALH	20 min pre-operation to the end of procedure	NM	LI-4,ST-36,PC-6	1. willingness to repeat the procedure
			G2: 51.5 ± 4.4	G2: TPALH	10 min pre-operation		Lidocaine gel 10 ml	
Yang (33)	China	200 (0)100/100	G1: 47.80 ± 14.68	G1: SFN + TPALH	15–20 min pre-operation to the end of procedure	Electronic gastroscope (Pentax)	ST-40;with hand manipulating for 2 min	1. INV
			G2: 48.60 ± 13.76	G2: SA + TPALH	Pre-operation		Lidocaine gel 10 ml	2. vas of discomfort
Qi and Jin (31)	China	102(0)51/51	G1: 50.74 ± 13.34	G1: AP + EA + T PALH	15 min pre-operation to the end of procedure	NM	ST-36, PC-6, TF4, AH6, CO4	1. vas of discomfort
			G2: 51.26 ± 13.15	G2: TPALH	10 min pre-operation		Lidocaine gel 10 ml	
Jin et al. (25)	China	102 (0)50/52	G1: 50.74 ± 13.35	G1: EA + TPALH	3–5 min pre-operation to the end of procedure	Electronic gastroscope	ST-36, PC-6	1. vas of discomfort
			G2: 51.27 ± 13.16	G2: SA + TPALH	Pre-operation		Lidocaine gel	
Dai et al. (39)	China	60 (0)30/30	G1: 49.03 ± 9.41	G1: MA + TPALH	5 min pre-operation to the end of procedure	Electronic gastroscope (Pentax)	ST-36, PC-6	1. INV
			G2: 52.14 ± 10.11	G2: TPALH	Pre-operation		Lidocaine gel	2. adverse effects
Liang et al. (36)	China	200 (0)100/100	G1: 17–69	G1: AP	10 min pre-operation to the end of procedure	Fibergastroscope	CO4, TG3, CO18; with hand manipulating for the whole procedure	1. INV
			G2: 16–70	G2: atropine 0.5 mg i.h + 1%dicaine for pharyngeal anesthesia	30 min pre-operation		Dicaine 1% spray three times	
Cahn et al. (16)	France	90 (0)45/45	NM	G1: EA	10 min pre-operation to the end of procedure	NM	ST-36, PC-6, SP-5, RN-23, RN-24, Shanzhong, RN-12	1. no. of intubation attempts
				G2: SA	NM		NM	2. eructation, vomiting attempts^a^, agitating & vomiting (E)
								3. pain in the pharynx, esophagus & stomach (P)
								4. nausea & bloating (P)
								5. willingness to repeat the procedure
Tarçin et al. (15)	Turkey	327 (14)78/79/79/77	48 ± 11 (range: 17–86)	G1: TENS + TPALH	15 min pre-operation to the end of procedure	Electrogastrography	PC-6	1. INV
				G2: sham-TENS + TPALH	Pre-operatin		Xylocaine 10 ml	2. willingness to repeat the procedure
				G3: sham-acupoints + TPALH				3. the swallowing scores;
				G4: no attachment + TPALH				4.the score of endoscopists' opinion regarded the procedure
Schaible et al. (21)	Germany	354 (0)177/177	G1:52.3 ± 13.5	G1: MA + TPALH	10 min pre-operation to the end of procedure	NM	RN-24, PC-6, LI-4	1. the frequency of successfully performed examination;
					Pre-operation		Xylocaine spray (AstraZeneca, Germany)	2. the duration of procedure;
			G2:53.4 ± 13.8	G2: SA + TPALH				3. willingness to repeat the procedure
								4. adverse effects
Leung et al. (34)	China	140 (0)70/70	NM	G1: MA	10 min pre-operation to the end of procedure	NM	HT-7,PC-6	1. vas of discomfort;
				G2: SA	NM		NM	2. adverse effects
								3. the anxiety scores
								4. the proportion of patients' graded overall tolerance as‘excellent or good'
								5. overall satisfaction scores

*INV, incidence of nausea and vomiting; TPALH, topical pharyngeal anesthesia with lidocaine hydrochloride; SA, sham acupuncture; SFN, superficial needle; AP, Auricular-Plaster; NM, not mentioned; adverse effects above were associated with acupuncture treatment.*

*Cahn et al. (16) study reported event rate of kinds of various discomfort symptom, among it some were assessed by endoscopist (E) and some were assessed by patients (P). Other studies reported the INV observed by the researcher and vas of discomfort evaluated by patients.*

*Schaible et al. (21) also reported other outcomes in the original paper, while considering that they were not out attention points, we did not present it here (e.g., heart rate; blood pressure, and oxygen saturation assessed at different time points: before esophagogastroduodenoscopy; after passage of the larynx; after removal of the endoscope)*.

**Table 2:** Reported outcomes of included studies.

**Studies**	**Outcomes**	**Sample size**	**Outcome measurement**	**Experiment group**	**Control group**	Difference*		
						**RR (95% CI)/MD**	***P*-value^**a**^**	***P*-value^**b**^**
**MA** **+** **TPALH V.S. TPALH**								
Wang et al. (28)	INV	60 30/30	ORR, *n* (%)	16 (53.33)	26 (86.67)	0.62 (0.43–0.88)	<0.050	=0.009
Zhou et al. (35)	INV	80 40/40	ORR, *n* (%)	31 (77.50)	36 (90.00)	0.86 (0.71–1.05)	<0.050	=0.140
Li and Wang (24)	willingness to repeat the procedure	98 49/49	ORR, *n* (%)	29 (59.18)	12 (24.49)	2.42 (1.40–4.16)	<0.050	=0.001
Dai et al. (39)	INV	60 30/30	ORR, *n* (%)	12 (40.00)	21 (70.00)	0.57 (0.35–0.94)	=0.019	=0.030
Wang (37)	vas of discomfort	108 54/54	Mean, SD	3.81 ± 1.48	4.71 ± 1.43	−0.90 (-1.45 to−0.35)	<0.050	=0.001
**MA V.S. TPALH**								
Zhang et al. (41)	INV	160 80/80	ORR, *n* (%)	40 (50.00)	40 (50.00)	1.00 (0.73–1.36)	<0.010	=1.000
Wang (40)	INV	300 169/131	ORR, *n* (%)	64 (37.87)	66 (50.38)	0.75 (0.58–0.97)	<0.050	=0.030
**MA V.S. SA**								
Leung et al. (34)	1.vas of discomfort	140 70/70	Mean, SD	1.60 ± 2.40	2.00 ± 2.70	−0.40 (-1.25, 0.45)	=0.391	=0.350
	2.adverse effects		Event rate	None	None	–	–	–
	3.the anxiety scores		Mean, SD	1.00 ± 2.40	1.10 ± 2.40	−0.10 (-0.90, 0.70)	=0.822	=0.810
	4.the proportion of patients' graded overall tolerance as‘excellent or good'		Event rate	36.00%	23.00%	–	=0.095	–
	5.overall satisfaction scores		mean, SD	8.10 ± 2.40	7.80 ± 2.20	0.30 (-0.46, 1.06)	=0.224	=0.440
**MA** **+** **TPALH V.S. SA** **+** **TPALH**								
Schaible et al. (21)	1.the frequency of successfully performed examination	354 177/177	event rate	73.50%	72.90%	–	=0.905	
	2.the duration of procedure		Average (min, max)	7 (2–20)	7 (2–25)	–	=0.406	
	3.willingness to repeat the procedure		Event rate	86.90%	87.60%	–	=0.857	
	4.adverse effects		Event rate	None	None	–	–	-
**MA V.S. no treatment**								
Tian and Wu (38)	INV	90 50/40	ORR, *n* (%)	32 (64.00)	38 (95.00)	0.67 (0.54–0.84)	<0.010	<0.001
**EA** **+** **TPALH vs. TPALH**								
Chen et al. (26)	1.INV	97 52/45	ORR, *n* (%)	21 (40.38)	41 (91.11)	0.44 (0.31–0.62)	<0.010	<0.001
	2.willingness to repeat the procedure		ORR, *n* (%)	24 (46.15)	3 (6.67)	6.92 (2.23–21.47)	<0.010	<0.001
Cui (22)	INV	137 66/68	ORR, *n* (%)	44 (66.67)	49 (70.06)	0.93 (0.74–1.16)	=0.045	=0.500
Zhou and Fang (23)	vas of discomfort	248 123/125	mean, SD	3.19 ± 2.29	4.28 ± 2.60	−1.09 (-1.71 to−0.47)	<0.050	<0.001
**EA V.S. SA**								
Cahn et al. (16).	1.no. of intubation attempts	90 45/45	-	-	-	-	EA < SA (*p* < 0.050)	–
	2.eructation, vomiting attempts^c^, agitating & vomiting (E)		Event rate				Ea < sa (*p* < 0.001) except not significant at 5% level in vomiting	1 = 0.002
	3.pain in the pharynx, esophagus & stomach (P)		Event rate				Pharynx: ea < sa (*p* < 0.010) esophagus:ea = sa stomach: ea < sa (*p* < 10^−6^)	–
	4.nausea & bloating (P)		Event rate				Nausea: ea < sa (*p* < 10^−4^) bloating: ea < sa (*p* < 0.050)	–
	5.willingness to repeat the procedure						EA = SA (not significant at 5% level)	=0.040
**EA** **+** **TPALH V.S.SA** **+** **TPALH**								
Jin et al. [25[	vas of discomfort	102 50/52	Mean, SD	3.82 ± 1.28	4.35 ± 1.40	−0.53 (-1.05 to−0.01)	<0.050	=0.050
**AP** **+** **TPALH V.S. TPALH**								
Qi (27)	1.vas of discomfort	80 40/40	Mean, SD	3.73 ± 1.32	4.33 ± 1.33	−0.60 (−1.18 to−0.02)	=0.046	=0.040
	2.willingness to repeat the procedure		ORR, *n* (%)	31 (77.50)	22 (55.00)	1.41 (1.02–1.95)	=0.033	=0.040
Liang et al. (36)	INV	200 100/100	ORR, *n* (%)	22 (22.00)	17 (17.00)	1.29 (0.73–2.29)	?	=0.370
**AP** **+** **EA** **+** **TPALH V.S. TPALH**								
Wu and Ye (32)	1.INV	100 50/50	ORR, *n* (%)	19 (38.00)	43 (86.00)	0.44 (0.30–0.64)	<0.050	<0.001
	2.willingness to repeat the procedure		ORR, *n* (%)	26 (52.00)	5 (10.00)	5.20 (2.17–12.45)	<0.010	<0.001
Qi and Jun (31)	vas of discomfort	102 51/51	Mean, SD	3.61 ± 1.43	4.51 ± 1.38	−0.90 (-1.45 to−0.35)	<0.050	=0.001
**SFN** **+** **TPALH V.S. SA** **+** **TPALH**								
Chen (29)	1.INV	60 30/30	ORR, *n* (%)	26 (86.67)	29 (96.67)	0.90 (0.77–1.05)	<0.010	=0.170
	2.vas of discomfort		Mean, SD	4.80 ± 1.65	6.30 ± 1.47	−1.50 (-2.29 to−0.71)	<0.010	<0.001
	3.willingness to repeat the procedure		ORR, *n* (%)	14 (46.67)	6 (20.00)	2.33 (1.04–5.25)	=0.028	=0.040
Yang (33)	1.INV	200 100/100	ORR, *n* (%)	32 (32.00)	66 (66.00)	0.48 (0.35–0.67)	<0.001	<0.001
	2.vas of discomfort		Mean, SD	2.94 ± 1.16	3.94 ± 1.15	−1.00 (-1.32 to−0.68)	<0.050	<0.001
**Acupressure** **+** **TPALH V.S. TPALH**								
Jiang (30)	INV	156 77/79	ORR, *n* (%)	54 (70.13)	69 (87.34)	0.80 (0.68–0.95)	<0.050	=0.010
**TENS** **+** **TPALH V.S. sham-TENS** **+** **TPALH V.S. sham-acupoints** **+** **TPALH V.S. no attachment** **+** **TPALH**								
Tarçin et al. (15)	1.INV	327 78/79/79/77	-	-	-	-	>0.005	
	2.willingness to repeat the procedure						>0.005	
	3.the swallowing scores						>0.050	
	4.the score of endoscopists' opinion regarded the procedure						>0.050	

*INV, incidence of nausea and vomiting incidence of nausea and vomiting incidence of nausea and vomiting; TPALH, topical pharyngeal anesthesia with lidocaine hydrochloride; SA, sham acupuncture; SFN, superficial needle; AP, Auricular-Plaster; ORR, overall response rate; adverse effects above were associated with acupuncture treatment.*

*We transformed ORRs into dichotomous variable (event rate), RR was calculated as event rate in experiment group divided by that in control group.*

^*^Changes of experiment and control group, mean difference (MD)/risk ratio (RR) and P-value^b^ were calculated based on data provided in the original papers using RevMan V.5.3. MD was calculated as mean difference of treatment effect (post-treatment-value minus control group value) in each comparison. P-value^a^ were data provided in the original papers (mean ± SD), mean means mean discomfort (VAS) score in each group measured right after upper GI endoscopy procedure.

*Cahn et al.'s (16) study reported event rate of kinds of various discomfort symptom, among it some were assessed by endoscopist (E) and some were assessed by patients (P). Other studies reported the INV observed by the researcher and vas of discomfort evaluated by patients.*

*Schaible et al. (21) also reported other outcomes in the original paper, while considering that they were not out attention points, we did not present it here (e.g., heart rate; blood pressure, and oxygen saturation assessed at different time points: before esophagogastroduodenoscopy; after passage of the larynx; after removal of the endoscope)*.

### Risk of Bias Assessment of Included Studies

**Figure 2:**
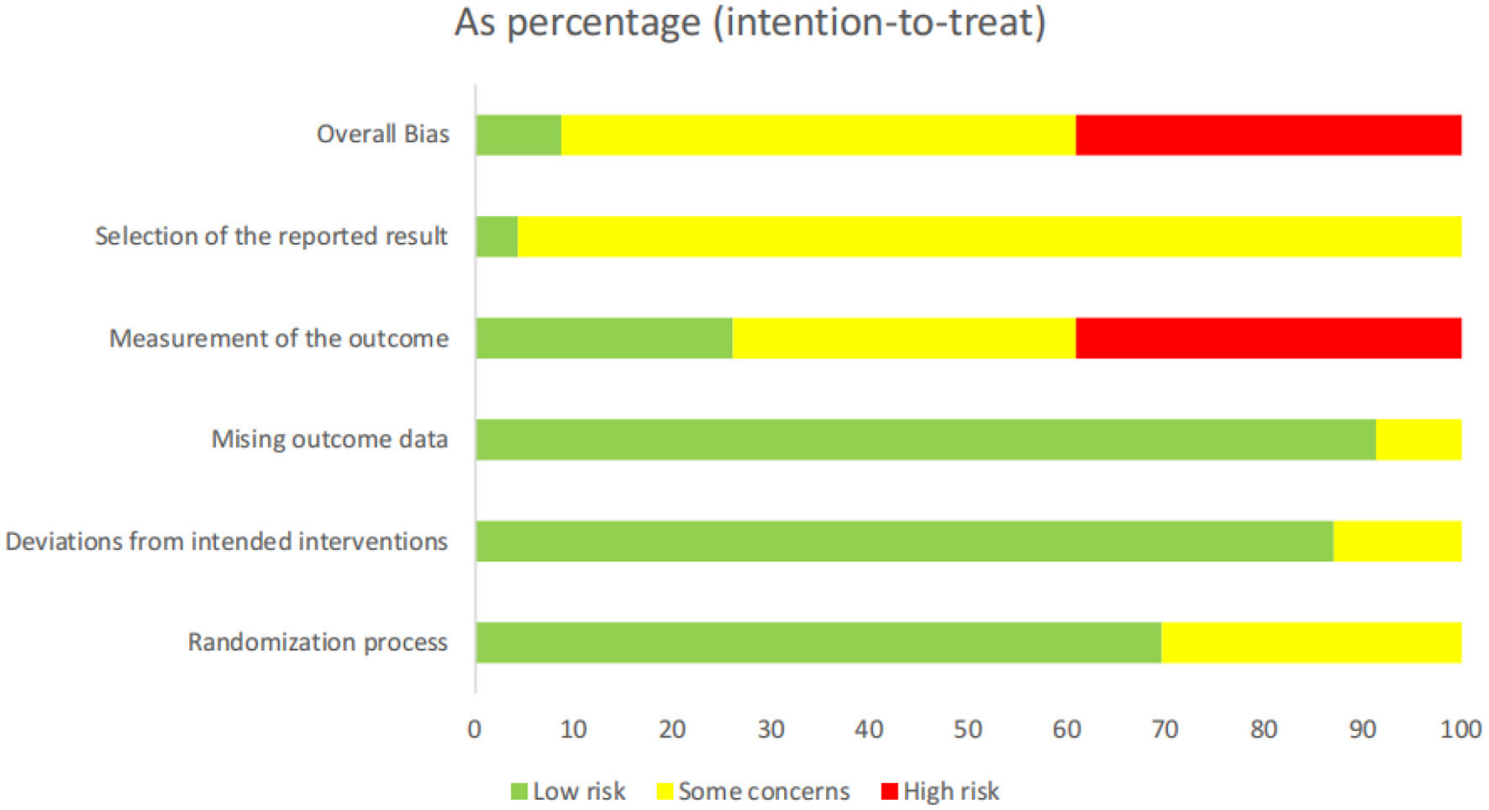
Risk of bias summary: review authors' judgement regarding each risk of bias item for each included study.

**Figure 3:**
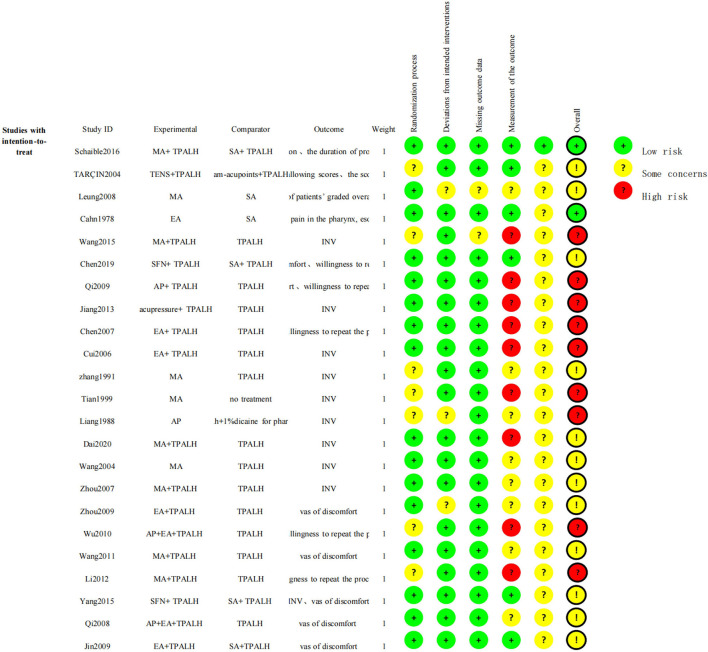
Methodological quality graph: review authors' judgements regarding each methodological quality item.

Most of the 23 studies had medium-to-high risk of bias, while one study had low risk of bias (21). Fourteen studies reported sound methods of random number generation, and nine studies did not contain detailed methods of randomization, while two studies out of nine were performed by experienced team which we assessed low risk of bias in randomization process. Five studies provided details regarding allocation concealment, while the rest did not. Six studies reported methods used for blinding patients and outcome assessors. Seventeen studies did not perform blinding of patients as their comparisons were between acupuncture and non-acupuncture treatment, and did not mention blinding of outcome assessors. Due to the characteristics of the acupuncture technique, doctors performing acupuncture treatment cannot be blinded. Almost all studies were considered low risk of attrition bias as the duration of intervention was short and no follow-up was conducted in any study other than two studies (28, 34). Except for a single study (21), the protocols were not available

to confirm whether the pre-designed outcomes were reported in their entirety (15, 16, 22–41). Two studies did not clarify whether baselines were comparable between different arms, and as such were considered to have other sources of bias (Figures 2, 3).

### Assessment of Effects - Manual Acupuncture

Ten studies (1,530 patients) investigated the effect of MA on improving discomfort among patients who underwent UGE. Among these studies, MA was compared with topical pharyngeal anesthesia with lidocaine hydrochloride (TPALH), sham-MA, usual care, and no treatment, with or without concomitant treatment. Two studies began MA and the UGE procedure at the same time (40, 41), while eight other studies began MA 3–20 min before the procedure and continued treatment until the end of the procedure (21, 24, 28, 34, 35, 37–41).

#### MA Plus TPALH vs. TPALH Alone

Five studies (406 patients) compared MA plus TPALH with TPALH alone. Dai et al. (39) and Zhou et al. (35) adopted the same acupuncture regimen (ST-36 and PC-6), while Wang (37) adopted ST-34, Wang et al. (28) adopted PC-6, and Li and Wang (24) adopted ST-36, PC-6 and LI-4.

**Figure 4:**
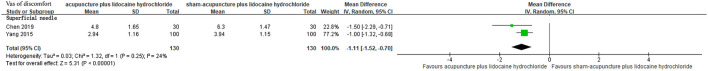
Forest plots of comparison between acupuncture plus lidocaine hydrochloride and sham acupuncture plus lidocaine hydrochloride.

A 2011 study by Wang reported that the VAS score of discomfort in the MA plus TPALH group was significantly lower compared to the TPALH alone group (3.81 ± 1.48 vs. 4.71 ± 1.43, MD −0.90, 95% CI −1.45 to −0.35, *P* = 0.001). In 2020, Dai reported significantly less INV in the MA plus TPALH group compared to the TPALH group (RR 0.57, 95% CI 0.35–0.94, *P* = 0.03), and Wang et al. (28) reported significantly less INV in the MA plus TPALH group that in the TPALH group (RR 0.62, 95% CI 0.43–0.88, *P* = 0.009). However, in a 2007 study by Zhou, the INV of each group (*P* < 0.05) was inconsistent with our calculation (RR 0.86, 95% CI 0.71–1.05, *P* = 0.14), in which we transformed the categorical data (overall effective rate) into dichotomous variables (event rate). Li and Wang (24) reported that the rate of patients willing to repeat the procedure in the MA plus TPALH group was 2.42 times higher compared to the TPALH-only group (RR 2.42, 95% CI 1.40–4.16; *P* = 0.001). By synthesizing the INV data from two studies (35, 39), it was determined that there was no significant difference between the MA plus TPALH and TPALH-only groups using a random-effect model (RR 0.74, 95% CI 0.47–1.15, *P* = 0.18, *I*^2^ = 66%, Figure 4).

#### MA vs. TPALH

Two studies (460 patients) investigated the effect of MA in comparison with TPALH but with varied timing of treatment and acupoints (40, 41). In 2004, Wang reported INV in the MA (PC-6) group was less than that in the TPALH group (RR 0.75, 95% CI 0.58–0.97; *P* = 0.03). In 1991, Zhang reported that INV was not statistically different between the two groups (PC-6, ST-36; RR 1.00, 95% CI 0.73–1.36; *P* = 1.00).

#### MA vs. Sham-MA

In 2008, a study by Leung (140 patients) compared MA to sham-MA, and reported that the VAS scores of discomfort (mainly pain) during the UGE were not significantly different between the two groups (1.6 ± 2.4 vs. 2.0 ± 2.7, MD −0.40, 95% CI −1.25 to 0.45, *P* = 0.35). This study also reported that there were no statistical differences regarding anxiety scores (MD −0.10, 95% CI −0.90 to 0.70, *P* = 0.81), the proportion of patients rating their overall tolerance as “excellent or good” (36 vs. 23%, *P* = 0.095), or the overall satisfaction scores (MD 0.30, 95% CI −0.46 to 1.06, *P* = 0.44) between the two groups.

Schaible et al. [(21); 354 patients] published a study comparing MA with sham-MA, where TPALH was used in both groups as standard care. This study reported that the rates of successfully performed UGE procedures (73.5 vs. 72.9%, *P* = 0.9045), as well as the proportions of patients willing to repeat the procedure (86.9 vs. 87.6%, *P* = 0.857), were not significantly different between the two groups. In addition, there were no significant differences in terms of heart rate, blood pressure, or oxygen saturation between the two groups at various time points (*P*-values were not provided). The percentage of patients with a reduced gag reflex was also not significantly different between the two groups (55.7 vs. 53.1%, *P* = 0.627).

#### MA vs. No Treatment

The 1999 Tian study (90 patients) compared the effect of MA with no treatment during UGE. The treatment effect was ranked as follows: (1) marked effective: mild discomfort in the epigastric area, without nausea or vomiting; (2) effective: moderate discomfort in the epigastric area, and the frequency of nausea and vomiting decreased to 1–3 times per minute; (3) ineffective: no improvement on symptoms of discomfort in the epigastric area, or nausea and vomiting. Overall response rate (ORR), the proportion of “marked effective” and “effective” cases, were used as the primary outcomes in this study. A significant difference of ORR between the MA and no treatment groups was found (90 vs. 47.5%, *P* < 0.01) given the baseline characteristics were comparable between the two groups.

### Assessment of Effects - Electroacupuncture

Five studies (674 patients) investigated the effect of EA on improvement of discomfort during UGE by comparing EA with TPALH or sham-EA, with or without concomitant treatment. The ST-36, LI-4, and PC-6 were used as principle acupoints in the regimens of these studies (16, 22, 23, 25, 26).

#### EA Plus TPALH vs. TPALH Alone

Three studies compared EA plus TPALH with TPALH alone. The ST-36 was used as the principle acupoint by all three studies (22, 23, 26).

A 2009 study by Zhou reported lower levels of VAS (discomfort) following treatment in the EA plus TPALH group compared to the TPALH-only group (3.19 ± 2.29 vs. 4.28 ± 2.6, MD −1.09, 95% CI −1.71 to −0.47, *P* = 0.0005). Chen et al. (26) reported INV was significantly lower in the EA plus TPALH group (RR 0.44, 95% CI 0.31–0.62; *P* < 0.00001). Cui reported in 2006 that the INV was significantly different between the two groups (*P* = 0.045), which was inconsistent with our calculation (RR 0.93, 95% CI 0.74–1.16; *P* = 0.50). Chen et al. (26) also reported the rate of patients willing to repeat the procedure in the EA plus TPALH group was approximately seven times higher than that in the control group (RR 6.92, 95% CI 2.23–21.47; *P* = 0.0008).

#### EA vs. Sham-EA

Two studies (192 patients) compared EA with sham-EA with or without TPALH as standard care (16, 25). In 2009, Jin reported the VAS score of discomfort in the EA plus TPALH group was significantly lower than that in the sham-EA plus TPALH group (3.82 ± 1.27 vs. 4.35 ± 1.40, *P* < 0.05), which was inconsistent with our calculation (MD −0.53, 95% CI −1.05 to −0.01; *P* = 0.05). Cahn et al. (16) reported that the incidences of eructation (*P* < 0.001), vomiting attempts (*P* < 0.001), and agitation (*P* < 0.001) assessed by the endoscopist were significantly lower in the experimental group, while the proportion of patients willing to repeat the procedure was not statistically different between the two groups (*P* > 0.05).

### Assessment of Effects - Auricular-Plaster

#### AP Plus TPALH vs. TPALH

Two studies (280 patients) investigated the effect of AP during UGE and reported conflicting results (27, 36). Qi (27) reported that the VAS score of discomfort in the AP plus TPALH group was lower than that in the TPALH-only group (3.73 ± 1.32, 4.33 ± 1.33, MD −0.60, 95% CI −1.18 to −0.02; *P* = 0.04), and the proportion of patients willing to repeat the procedure was also higher in the AP plus TPALH group (RR 1.41, 95% CI 1.02–1.95; *P* = 0.04). On the contrary, Liang reported in 1988 that the INV in the AP group was higher than that in the atropine plus dicaine group (RR 1.29, 95% CI 0.73–2.29; *P* = 0.37).

#### AP Plus EA & TPALH vs. TPALH

Two studies (202 patients) compared the effect of AP plus EA and TPALH with TPALH alone (31, 32). The regimens and schedule of interventions were similar between the two studies. In 2008, Qi reported that the AP plus EA and TPALH group had significantly lower VAS scores of discomfort compared with the TPALH-only group (3.61 ± 1.43 vs. 4.51 ± 1.38, MD −0.90, 95% CI −1.45 to −0.35; *P* = 0.001), and the results of the 2010 Wu study on INV (RR 0.44, 95% CI 0.30–0.64; *P* < 0.0001) and the proportion of patients willing to repeat the procedure (RR 5.20, 95% CI 2.17–12.45; *P* < 0.0002) supported better outcomes in the AP plus EA and TPALH group compared with that of the TPALH-only group.

### Assessment of Effects - Superficial Needle

#### SFN Plus TPALH vs. Sham-SFN Plus TPALH

Two studies (260 patients) compared SFN plus TPALH with sham-SFN plus TPALH for discomfort during UGE (29, 33). Yang (33) reported that the experimental group was more effective in reducing INV (RR 0.48, 95% CI 0.35–0.67; *P* < 0.00001), while Chen (29) did not find a significant difference in INV between the two groups (RR 0.90, 95% CI 0.77–1.05, *P* = 0.17). For VAS scores of discomfort, both studies found that the SFN plus TPALH group showed greater improvement compared to control [Yang (33): MD −1.00, 95% CI −1.32 to −0.68, *P* < 0.00001; Chen (29): MD −1.50, 95% CI −2.29 to −0.71, *P* = 0.0002]. Chen (29) also reported the proportion of patients willing to repeat the procedure in the SFN group was higher than that in the control group (RR 2.33, 95% CI 1.04–5.25, *P* = 0.04).

**Figure 5:**
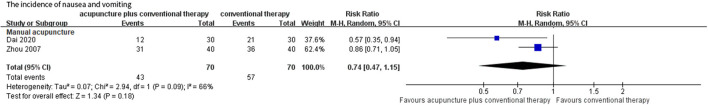
Forest plots of comparison between acupuncture plus lidocaine hydrochloride and lidocaine hydrochloride.

The 2019 Chen study used VAS to primarily measure the feeling of pain, while the 2015 Yang study measured general discomfort during the UGE procedure. Considering that pain carries considerable weight in discomfort, the VAS score data of the two studies were combined. These new results revealed that patients receiving SFN plus TPALH reported a greater improvement on the VAS scores of discomfort compared to sham-SFN plus TPALH group using a random-effect model (MD −1.11, 95% CI −1.52 to −0.70, *P* < 0.00001; *I*^2^ = 24%, Figure 5).

### Assessment of Effects - Acupressure

A single 2013 study by Jiang (156 patients) compared acupressure plus TPALH to TPALH alone (30). The study reported that patients in the experimental group had a lower INV compared to the control group (RR 0.80, 95% CI 0.68–0.95, *P* = 0.01).

### Assessment of Effects - Transcutaneous Electrical Nerve Stimulation

One study by Tarçin et al. (327 patients) designed a four-arm study, comparing the effects of TENS plus TPALH, sham-TENS plus TPALH, sham-acupoints plus TPALH, and standard care with TPALH alone to assess discomfort during UGE (15). PC-6 was used as the acupoint of stimulation. As reported, there were no significant differences found between the groups on nausea-retching scores (*P* > 0.05), swallowing scores (*P* > 0.005), score of the endoscopists' opinion of the procedure (*P* > 0.005), and the proportion of patients who would accept re-endoscopy (*P* > 0.05).

### Adverse Events

Among the 23 studies, four studies (17.39%) reported that there were no adverse events associated with acupuncture. One study (4.35%) reported that a single patient in the EA group could not complete the UGE procedure due to discomfort. The remaining 18 studies (78.26%) did not report any adverse events.

### Publication Bias

Funnel plots and Egger's test were not feasible due to the limited number of studies included for each type of intervention in the review (42).

## Discussion

To the best of our knowledge, this is the first systematic review and meta-analysis to assess the effect of acupuncture on the improvement of discomfort during UGE procedures. Among the 23 included RCTs, the results (improvement of VAS or INV) were in favor of acupuncture plus TPA (primarily TPALH) compared with TPA alone, among studies of MA, EA, AP, SFN, and acupressure. However, the results appeared inconsistent when comparing acupuncture methods alone to anesthetics, sham acupuncture, usual care, or no treatment. Most of the included studies did not report any adverse events in their findings and were of medium-to-high risk of bias.

Some studies explored the anti-emetic effects of acupuncture that might be associated with an increase in the hypophyseal secretion of beta-endorphins and adrenocorticotropic hormone, together with subsequent suppression of the chemoreceptor trigger zone and vomiting center (43, 44). Studies have shown that the Neiguan (PC-6) acupoint, which is the most commonly used acupoint to treat GI symptoms, may reduce nausea through a variety of mechanisms, including neurotransmitters (e.g., the endogenous opioid system, serotonin transmission), a direct influence on the smooth muscle of the gut, somatovisceral reflex, sensory input inhibition, somatosympathetic reflex-induced gastric relaxation, vagal modulation, central cerebellar modulation, or psychological aspects (45, 46). Some studies have indicated that Zusanli (ST-36) and Neiguan (PC-6) have a synergistic effect on gastric myoelectrical activity (47, 48). However, the true mechanism by which acupuncture relieves discomfort during UGE remains inconclusive.

An early systematic review (2004) (17) on discomfort during GI endoscopy (including UGE and colonoscopy) with six RCTs found that the effect of acupuncture (EA and MA) on relieving discomfort was similar to active medication, but better than that of sham acupuncture, with or without TPA or a sedative (17). The results described in the current review suggest that regardless of the type of acupuncture, the VAS score of discomfort during UGE in groups with acupuncture plus TPA was significantly lower than of TPA-alone, which was not claimed in the previous review. These results could indicate that the use of acupuncture as an adjuvant therapy could enhance the effect of TPA and thus may reduce the amount of TPA required during UGE. When comparing EA or MA with sham acupuncture, the results in the current review were inconsistent across studies with or without TPA, which diverged from the conclusions of the previous study (17).

It was unfortunate that we did not find substantiative evidence regarding the minimum clinical important difference of the VAS scores of discomfort (one of the major measurements of discomfort) during GI endoscopy from previous studies and systematic reviews (17). The discomfort during UGE and colonoscopy procedures is often regarded as comparable due to one common mechanism - the pressure of air distension (49). One previous study reported that the VAS pain scores during a colonoscopy were significantly lower in patients receiving anesthetics plus acupuncture (1.4 ± 0.4) compared to patients receiving anesthetics plus sham acupuncture (3.0 ± 0.3), with a difference of −1.30 (−1.58, −1.02, *P* = 0.003) (50). Another study reported the VAS discomfort scores in EA and SA groups were significantly different at 24 mmHg (pressure of air distension) during a colonoscope (2.80 vs. 4.74, *P* = 0.013) (51). In the current review, the mean VAS scores of discomfort ranged from 2.94 to 4.80 after treatment in patients receiving TPA plus acupuncture, and from 3.94 to 6.3 in patients receiving TPA only, with MD ranging from −1.11 to −0.65 (*P* all <0.05). Although the data of the VAS scores presented above looks comparable across studies, it would be arbitrary to draw any conclusion with the limited data and substantial heterogeneity regarding type of acupuncture, regimens (including beginning and ending time of acupuncture treatment in relation to the endoscopy, the total duration of endoscopy, acupoints selected, intensity of simulation), skills of doctors, as well as level of risk of bias.

It is worth mentioning that the outcome measurements used by the studies included in this review varied considerably, which hindered the syntheses of effects across all studies. For instance, considering the level of discomfort, nearly half of the included studies did not use internationally recognized tools, such as VAS or NRS, to measure the level of discomfort. Instead, they developed a ranking system to categorize the effect of acupuncture without a consistent definition for each rank across multiple studies. In addition, numerous factors can influence the discomfort level during a UGE procedure, such as the size of endoscopy lens, physical sensitivity and characteristics of the patients (e.g., age, sex, tolerance, upper gastrointestinal diseases, and previous endoscopy experience), time of measurement, the UGE operator's experience (52), etc. However, limited information was reported on the above factors to allow for further understanding or analysis on the effect of acupuncture. Given the side effect of TPA or sedatives, a reduced dosage when combined with acupuncture should be another key reflection of the effect of acupuncture. However, not all studies reported on this outcome (28, 35). None of the studies reported any data on cost-effectiveness of the use of acupuncture during UGE.

**Figure 6:**
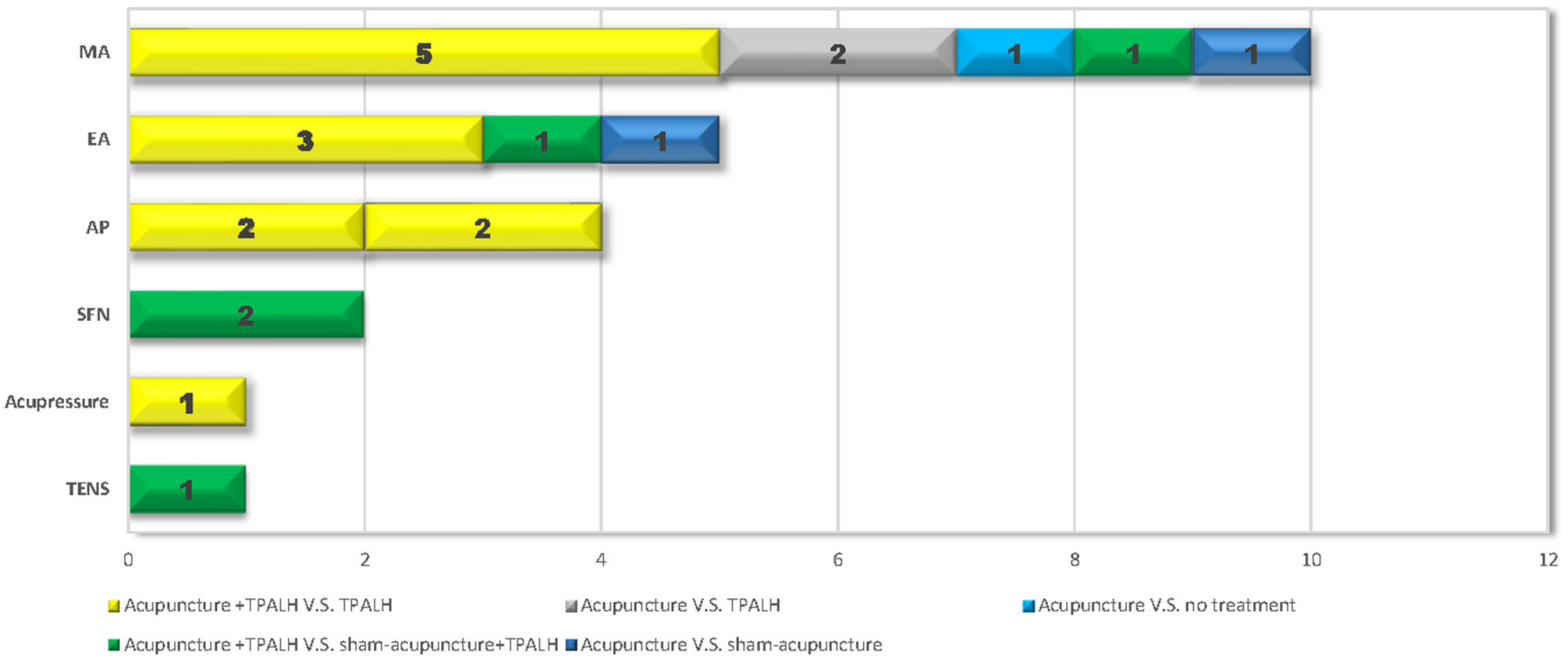
Categories of included studies.

The current review has many strengths. It included a greater number of studies than the previous review, focused specifically on unsedated UGE, and used a comprehensive search of both English and Chinese language biomedical databases. However, several limitations are also present. Firstly, the 23 RCTs were heterogeneous regarding the type and regimen of acupuncture and the control group, as well as outcome measurements, which limited our attempt to synthesize the effect from individual studies (Figure 6). Secondly, the tolerance of discomfort and acceptance of sedated UGE vary considerably among patients in different countries. More than 90% (21/23) of the included studies were conducted in Asian countries (i.e., China, Turkey), and only two were carried out in Europe (i.e., France, Germany), which may constrain the generalization of the results. Thirdly, with limited information, the review was not able to determine the specific characteristics of patients (e.g., sex, age) may benefit more from acupuncture, and which type of acupuncture and stimulation were superior to others. Fourthly, due to the lauguage capacity, we didnot search Korean or Japanese databases specially, which might add publication bias out of regional inequality.

## Conclusions

In this review, it was shown that acupuncture, as an adjuvant therapy to TPA, may further decrease discomfort levels compared to TPA alone. When compared with sham acupuncture, usual care, or no treatment, the effect of acupuncture was not consistent. Findings from this review should be interpreted with caution given the heterogeneity and bias identified across the studies. Rigorously designed RCTs that measure standardized and clinically relevant outcomes are needed to inform clinical decision-making regarding the use of acupuncture for discomfort relief during unsedated UGE procedures.

## Data Availability Statement

The original contributions presented in the study are included in the article/Supplementary Material, further inquiries can be directed to the corresponding author/s.

## Author Contributions

WW and ZL contributed to study conception and design. NG and HC searched the databases, reviewed studies, and assessed the quality of studies. YW and YG analyzed data and carried out the statistical analysis. This manuscript was drafted by NG and revised by WW and HC. All authors approved the final version of the manuscript.

## Funding

This study was funded by the China Academy of Chinese Medical Sciences (Grant No. ZZ13-YQ-019). The funding agency had no role in the design or conduct of the study.

## Conflict of Interest

The authors declare that the research was conducted in the absence of any commercial or financial relationships that could be construed as a potential conflict of interest.

## Publisher's Note

All claims expressed in this article are solely those of the authors and do not necessarily represent those of their affiliated organizations, or those of the publisher, the editors and the reviewers. Any product that may be evaluated in this article, or claim that may be made by its manufacturer, is not guaranteed or endorsed by the publisher.

## Abbreviations

UGE: upper gastrointestinal endoscopy
RCTs: randomized controlled trials
TPALH: topical pharyngeal anesthesia with lidocaine hydrochloride
VAS: visual analog scale
INV: incidence of nausea and vomiting
SFN: superficial needle
TPA: topical pharyngeal anesthesia
GI: gastrointestinal
PRISMA: Preferred Reporting Items for Systematic Reviews and Meta-Analyses
NRS: numerical rating scale
MD: mean difference
RR: risk ratio
Cis: confidence intervals
SA: sham acupuncture
AP: auricular plaster
EA: electroacupuncture
MA: manual acupuncture
TENS: transcutaneous electric nerve stimulation
ORR: overall response rate.
