# Supplementary material for: Acupuncture to Improve Patient Discomfort During Upper Gastrointestinal Endoscopy: Systematic Review and Meta-Analysis
Source: Front Med (Lausanne). 2022 Jun 3;9:865035. doi: 10.3389/fmed.2022.865035 (PMC9204029; doi:10.3389/fmed.2022.865035)
Supplement: Supplementary file 1 [file Table_1.pdf]

APPENDIX

Search Strategies

**MEDLINE**			total
1#	randomized controlled trial.pt.	516343	299
2#	controlled clinical trial.pt.	93912	
3#	randomized.ab.	497574	
4#	placebo.ab.	212224	
5#	randomly.ab.	344066	
6#	trial.ab.	525929	
7#	groups.ab.	2111284	
8#	drug therapy.fs.	2248164	
9#	1 or 2 or 3 or 4 or 5 or 6 or 7 or 8	4830128	
10#	exp Animals/ not humans.sh.	4752348	
11#	exp Acupuncture Points/ or exp Acupuncture, Ear/ or exp Acupuncture/ or exp Acupuncture Therapy/ or exp Electroacupuncture/ or exp Electric Stimulation Therapy/ or exp Acupressure/ or exp moxibustion/	102195	
12#	(acupuncture$ or "fire needling" or "warm needling" or pyonex or "intradermal needling").ab,ti.	22472	
13#	("gastroscop$" or "endoscopy" or "endoscope").ab,ti.	79788	
14#	("superior gastrointestinal endoscope" or "upper endoscope" or "upper gastrointestinal endoscope" or "uppergastrointestinal endoscope" or "digestive endoscope").ab,ti.	125	
15#	exp Gastroscopy/	17093	
16#	exp Gastroscopes/	1072	
17#	exp Endoscopy/ or exp Endoscopy, Gastrointestinal/ or exp Endoscopy, Digestive System/	356281	
18#	exp Endoscopes/	24706	
19#	11 or 12	107568	
20#	13 or 14 or 15 or 16 or 17 or 18	400405	
21#	9 not 10	4195030	
22#	19 and 20 and 21	299	

**EMBASE**			total
1#	randomized controlled trial/	631591	232
2#	controlled clinical trial/	466346	
3#	randomization/	89020	
4#	intermethod comparison/	267478	
5#	random$.ab,ti.	1600966	
6	placebo.ab,ti.	315002	
7	(compare or compared or comparison).ti.	524669	
8	(evaluated or evaluate or evaluating or assessed or assess).mp. and (compare or compared or comparing	2221556	
9	(open adj label).ab,ti.	82932	
10	((double or single or doubly or singly) adj (blind or blinded or blindly)).ab,ti.	238515	
11	double blind procedure/	178494	
12	parallel group$1.ab,ti.	26504	
13	(crossover or cross over).ab,ti.	108157	

14	((assign$ or match or matched or allocation) adj5 (alternate or group$1 or intervention$1 or patient$1 or	342150
15	(assigned or allocated).ab,ti.	403281
16	(controlled adj7 (study or design or trial)).ab,ti.	363479
17	(volunteer or volunteers).ab,ti.	253452
18	human experiment/	525072
19	trial.ti.	314552
20	or/1-19	5218365
21	(random$ adj sampl$ adj7 ("cross section$" or questionnaire$1 or survey$ or database$1)).ab,ti. not	
22	cross-sectional study/ not (randomized controlled trial/ or controlled clinical study/ or controlled study/	251772
23	(((case adj control$) and random$) not randomi?ed controlled).ab,ti.	17853
24	(Systematic review not (trial or study)).ti.	158245
25	(nonrandom$ not random$).ab,ti.	16594
26	"Random field$".ab,ti.	2440
27	(random cluster adj3 sampl$).ab,ti.	1319
28	(review.ab. and review.pt.) not trial.ti.	844279
29	"we searched".ab. and (review.ti. or review.pt.)	34099
30	"update review".ab.	112
31	(databases adj4 searched).ab.	38870
32	(rat or rats or mouse or mice or swine or porcine or murine or sheep or lambs or pigs or piglets or rabbit	1090174
33	animal experiment/ not (human experiment/ or human/)	2294518
34	or/21-33	3590696
35	20 not 34	4641163
36	exp auricular acupuncture/ or exp acupuncture/ or exp acupuncture point/ or exp acupuncture needle/ or	48297
37	exp electroacupuncture/	6973
38	exp moxibustion/	3128
39	exp acupressure/	2299
40	(acupuncture$ or "fire needling" or "warm needling" or pyonex or "intradermal needling").ab,ti.	31924
41	36 or 37 or 38 or 39 or 40	50998
42	("gastroscop$" or "endoscopy" or "endoscope").ab,ti.	137324
43	("superior gastrointestinal endoscope" or "upper endoscope" or "upper gastrointestinal endoscope" or "uppergastrointestinal endoscope" or "digestive endoscope").ab,ti.	358
44	exp gastroscopy/	22228
45	exp gastroscope/	3109
46	exp digestive tract endoscopy/ or exp gastrointestinal endoscopy/ or exp endoscopy/	643411
47	exp endoscope/	64250
48	42 or 43 or 44 or 45 or 46 or 47	694948
49	35 and 41 and 48	232

**the Cochrane Central Register of Controlled Trials (CENTRAL)**		total
#1 MeSH descriptor: [Acupuncture Therapy] explode all trees MeSH	4733	941
#2 (acupuncture or needl*):ti,ab,kw(Word variations have been searched)S Limits	29043	
#3 MeSH descriptor: [Transcutaneous Electric Nerve Stimulation] explode all trees	1900	
#4 (controlled clinical trial):pt(Word variations have been searched)S Limits	323446	
#5 ("Randomised" or "Randomly" or "Placebo" or "Trial" or "trail groups"):ti,ab,kw(Word variations have been searched)S Limits	1214589	
#6 MeSH descriptor: [Double-Blind Method] explode all trees MeSH	138084	

#7 MeSH descriptor: [Single-Blind Method] explode all trees MeSH	20895
#8 MeSH descriptor: [Random Allocation] explode all trees MeSH	20605
#9 #4or#5or#6or#7or#8 Limits	1302196
#10 MeSH descriptor: [Acupuncture] explode all trees MeSH	151
#11 ("gastroscop*" or "endoscopy" or "endoscope"):ti,ab,kw(Word variations have been searched)S Limits	26486
#12 ("superior gastrointestinal endoscope" or "upper endoscope" or "upper gastrointestinal endoscope" or "uppergastrointestinal endoscope"	131
#13 MeSH descriptor: [Gastroscopy] explode all trees MeSH	842
#14 MeSH descriptor: [Gastroscopes] explode all trees MeSH	318
#15 #1or#2or#3or#10 Limits	31056
#16 MeSH descriptor: [Endoscopy, Digestive System] explode all trees MeSH	5310
#17 MeSH descriptor: [Endoscopes, Gastrointestinal] explode all trees MeSH	266
#18 #11or#12or#13or#14or#16or#17 Limits	28133
#19 #9and#15and#18 Limits	941

**Scopus**		total
1 TITLE-ABS-KEY ("randomized controlled trial" OR "controlled clinical trial" OR randomized OR placebo OR randomly OR trial OR groups)	10,449,313	180
2 TITLE-ABS-KEY ("Acupuncture therapy" OR "Acupuncture" OR "electroacupuncture" OR "fire needling" OR "Acupressure" OR moxibustion OR "Electric Stimulation Therapy" OR "acupoint*" OR "warm needling" OR "pyonex" OR "intradermal needling")	69,903	
3 TITLE-ABS-KEY ("superior gastrointestinal endoscope" OR "upper endoscope" OR "upper gastrointestinal endoscope" OR "uppergastrointestinal endoscope" OR "digestive endoscope" OR "gastroscop*" OR "endoscopy" OR "endoscope")	241,442	
4 #1 AND #2 AND #3	180	

**Web of science**	
TS=("randomized controlled trial" OR "controlled clinical trial" OR randomized OR placebo OR randomly OR trial OR groups) AND (TS=("electroacupuncture" OR "acupoint*" OR "acupuncture therapy" OR “transcutaneous electric nerve stimulation” OR “acupuncture” OR "ear acupuncture" OR “acupressure” OR “moxibustion” OR "fire needling" OR "warm needling" OR pyonex OR "intradermal needling” OR "electric stimulation therapy")) AND (TS=("gastroscop*" OR "endoscopy" OR "endoscope" OR " superior gastrointestinal endoscope " OR “upper endoscope” OR “upper gastrointestinal endoscope” OR “uppergastrointestinal endoscope” OR “digestive endoscope”)	22

**Chinese BioMedical Literature Database (CBM)**	
1 "上消化道内镜"[常用字段:智能] OR "消化道内镜"[常用字段:智能] 770	237
2 "胃镜检查"[不加权:扩展] OR "胃镜"[不加权:扩展]	24517
3 "针灸"[常用字段:智能] OR "针刺"[常用字段:智能] OR "艾灸"[常用字段:智能] OR "物理疗法"[常用字段:智能] OR "电针"[常用字段:智能] OR "耳针"[常用字段:智能] OR "体针"[常用字段:智能] OR "腕踝针"[常用字段:智能] OR "拔罐"[常用字段:智能]	191959
4 ("针灸疗法"[不加权:扩展]) OR "针刺疗法"[不加权:扩展] OR "针刺"[不加权:扩展]	166803
5 (#1) OR (#2)	25217
6 (#3) OR (#4)	230177
7 (#5) AND (#6)	237

The Chinese Science and Technology Journal Full-text Database (CNKI)	
SU=('胃镜'+'上消化道内镜'+'内镜'+'消化道内镜')*('针灸'+'针刺'+'艾灸'+'物理疗法'+'电针'+'电针'+'耳针'+'体针'+'腕踝针'+'拔罐')	346

Wan Fang Data	
TI=(“胃镜”OR”上消化道内镜”OR”内镜”OR”消化道内镜”) AND(“针灸”OR”针刺”OR”艾灸”OR”物理疗法”OR”电针”OR”电针”OR”耳针”OR”体针”OR”腕踝针”OR”拔罐”)	155

The Chinese Biomedical Literature Database (VIP)	
M=(胃镜 OR 上消化道内镜 OR 内镜 OR 消化道内镜) AND M=(针灸 OR 针刺 OR 艾灸 OR 物理疗法 OR 电针 OR 电针 OR 耳针 OR 体针 OR 腕踝针 OR 拔罐)	50
