# Supplementary material for: Acupuncture to Improve Patient Discomfort During Upper Gastrointestinal Endoscopy: Systematic Review and Meta-Analysis
Source: Front Med (Lausanne). 2022 Jun 3;9:865035. doi: 10.3389/fmed.2022.865035 (PMC9204029; doi:10.3389/fmed.2022.865035)
Supplement: Supplementary file 2 [file Presentation_1.PDF]

Acupuncture plus lidocaine hydrochloride compared to lidocaine hydrochloride for discomfort in patients during upper gastrointestinal endoscopy

Patient or population: discomfort in patients during upper gastrointestinal endoscopy
Setting: outpatients/inpatients
Intervention: Acupuncture plus lidocaine hydrochloride
Comparison: lidocaine hydrochloride

Outcomes	№ of participants (studies) Follow-up	Certainty of the evidence (GRADE)	Relative effect (95% CI)	Anticipated absolute effects	
				Risk with lidocaine hydrochloride	Risk difference with Acupuncture plus lidocaine hydrochloride
the incidence of vomiting and nausea	140 (2 RCTs)	⨁◯◯◯ Very low[a,b,c]	**RR 0.74** (0.47 to 1.15)	850 per 100	**221 fewer per 100** (451 fewer to 127 more)

***The risk in the intervention group** (and its 95% confidence interval) is based on the assumed risk in the comparison group and the **relative effect** of the intervention (and its 95% CI).

CI: confidence interval; **RR:** risk ratio

GRADE Working Group grades of evidence
High certainty: we are very confident that the true effect lies close to that of the estimate of the effect.
Moderate certainty: we are moderately confident in the effect estimate: the true effect is likely to be close to the estimate of the effect, but there is a possibility that it is substantially different.
Low certainty: our confidence in the effect estimate is limited: the true effect may be substantially different from the estimate of the effect.
Very low certainty: we have very little confidence in the effect estimate: the true effect is likely to be substantially different from the estimate of effect.

Explanations

a. The sample size is small (total number of events <300).
b. The unclear risk of selection bias due to the incomplete reporting of concealment of allocation, the high risk of detection and performance bias due to the unblinding of the participants/ personnel and outcome assessors.
c. We downgraded the certainty of the evidence once for inconsistency; The pooled effects estimates showed considerable heterogeneity.
