# Supplementary material for: Acupuncture to Improve Patient Discomfort During Upper Gastrointestinal Endoscopy: Systematic Review and Meta-Analysis
Source: Front Med (Lausanne). 2022 Jun 3;9:865035. doi: 10.3389/fmed.2022.865035 (PMC9204029; doi:10.3389/fmed.2022.865035)
Supplement: Supplementary file 3 [file Presentation_2.PDF]

Acupuncture plus lidocaine hydrochloride v.s. Sham-acupuncture plus lidocaine hydrochloride for discomfort in patients during upper gastrointestinal endoscopy

Patient or population: discomfort in patients during upper gastrointestinal endoscopy
Setting: outpatients/inpatients
Intervention: Acupuncture plus lidocaine hydrochloride
Comparison: Sham-acupuncture plus lidocaine hydrochloride

Outcomes	№ of participants (studies) Follow-up	Certainty of the evidence (GRADE)	Relative effect (95% CI)	Anticipated absolute effects	
				Risk with Sham-acupuncture plus lidocaine hydrochloride	Risk difference with Acupuncture plus lidocaine hydrochloride
vas of discomfort	260 (2 RCTs)	⨁⨁◯◯ Low[a,b]	-		MD **1.11 lower** (1.52 lower to 0.7 lower)

***The risk in the intervention group** (and its 95% confidence interval) is based on the assumed risk in the comparison group and the **relative effect** of the intervention (and its 95% CI).

CI: confidence interval; **MD:** mean difference

GRADE Working Group grades of evidence
High certainty: we are very confident that the true effect lies close to that of the estimate of the effect.
Moderate certainty: we are moderately confident in the effect estimate: the true effect is likely to be close to the estimate of the effect, but there is a possibility that it is substantially different.
Low certainty: our confidence in the effect estimate is limited: the true effect may be substantially different from the estimate of the effect.
Very low certainty: we have very little confidence in the effect estimate: the true effect is likely to be substantially different from the estimate of effect.

Explanations

a. The unclear risk of selection bias due to the incomplete reporting of concealment of allocation, the high risk of detection and performance bias due to the unblinding of the participants/ personnel and outcome assessors.
b. The sample size is small (total number of events <300).
